# 2,4-Dimethyl­phenyl 4-methyl­benzoate

**DOI:** 10.1107/S1600536809039397

**Published:** 2009-10-03

**Authors:** B. Thimme Gowda, Miroslav Tokarčík, Jozef Kožíšek, P. A. Suchetan, Hartmut Fuess

**Affiliations:** a Department of Chemistry, Mangalore University, Mangalagangotri 574 199, Mangalore, India; b Faculty of Chemical and Food Technology, Slovak Technical University, Radlinského 9, SK-812 37 Bratislava, Slovak Republic; c Institute of Materials Science, Darmstadt University of Technology, Petersenstrasse 23, D-64287 Darmstadt, Germany

## Abstract

In the title compound, C_16_H_16_O_2_, the two aromatic rings form a dihedral angle of 49.1 (1)°. In the crystal structure, there are no classical hydrogen bonds. The long axes of the mol­ecules are directed along the *c* axis.

## Related literature

For the preparation of the compound, see: Nayak & Gowda (2009 ▶). For background to our study of the effect of substituents on the crystal structures of aryl benzoates and for related structures, see: Gowda, Foro *et al.* (2007 ▶, 2008 ▶); Gowda, Tokarčík *et al.* (2008 ▶, 2009 ▶). For phenyl benzoate, see: Adams & Morsi (1976 ▶);
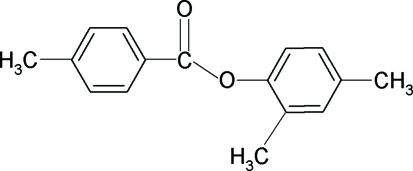


## Experimental

### 

#### Crystal data

C_16_H_16_O_2_
*M* *_r_* = 240.29
Monoclinic, 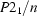

*a* = 11.8022 (3) Å
*b* = 7.4959 (2) Å
*c* = 15.6288 (4) Å
β = 107.760 (3)°
*V* = 1316.75 (6) Å^3^
*Z* = 4
Mo *K*α radiation
μ = 0.08 mm^−1^
*T* = 295 K
0.52 × 0.38 × 0.12 mm

#### Data collection

Oxford Diffraction Xcalibur2 diffractometer with a Sapphire CCD detector
Absorption correction: multi-scan (*CrysAlis RED*; Oxford Diffraction, 2009 ▶) *T*_min_ = 0.96, *T*_max_ = 0.991
15897 measured reflections
2497 independent reflections
1917 reflections with *I* > 2σ(*I*)
*R*_int_ = 0.018

#### Refinement

*R*[*F* ^2^ > 2σ(*F* ^2^)] = 0.042
*wR*(*F* ^2^) = 0.126
*S* = 1.09
2497 reflections
167 parameters
H-atom parameters constrained
Δρ_max_ = 0.15 e Å^−3^
Δρ_min_ = −0.14 e Å^−3^

### 

Data collection: *CrysAlis CCD* (Oxford Diffraction, 2009 ▶); cell refinement: *CrysAlis RED* (Oxford Diffraction, 2009 ▶); data reduction: *CrysAlis RED*; program(s) used to solve structure: *SHELXS97* (Sheldrick, 2008 ▶); program(s) used to refine structure: *SHELXL97* (Sheldrick, 2008 ▶); molecular graphics: *ORTEP-3* (Farrugia, 1997 ▶) and *DIAMOND* (Brandenburg, 2002 ▶); software used to prepare material for publication: *SHELXL97*, *PLATON* (Spek, 2009 ▶) and *WinGX* (Farrugia, 1999 ▶).

## Supplementary Material

Crystal structure: contains datablocks I, global. DOI: 10.1107/S1600536809039397/bt5076sup1.cif

Structure factors: contains datablocks I. DOI: 10.1107/S1600536809039397/bt5076Isup2.hkl

Additional supplementary materials:  crystallographic information; 3D view; checkCIF report

## Figures and Tables

**Fig. 1.**
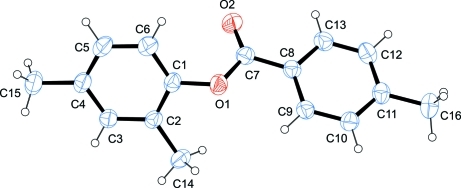
Molecular structure of the title compound showing the atom labelling scheme. Displacement ellipsoids are drawn at the 30% probability level and H atoms are represented as small spheres of arbitrary radii.

**Fig. 2.**
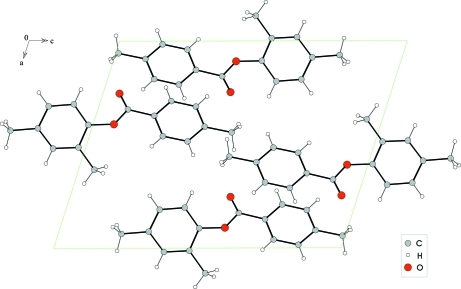
Molecular packing of the title compound as viewed along the b-axis.

## supplementary crystallographic information

### Comment

As part of studying the substituent effects on the crystal structures of aryl benzoates (Gowda, Foro *et al.*, 2007; 2008; Gowda, Tokarčík *et al.*, 2008; 2009), the structure of 2,4-dimethylphenyl 4-methylbenzoate (I) has been determined. The structure of (I) (Fig. 1) is similar to those of phenyl benzoate (II)(Adams & Morsi, 1976), phenyl 4-methylbenzoate (III) (Gowda, Tokarčík *et al.*, 2009), 2-methylphenyl 4-methylbenzoate (IV) (Gowda, Foro *et al.*, 2008), 4-methylphenyl 4-methylbenzoate (V) (Gowda, Foro *et al.*, 2007) and 2,4-dimethylphenyl benzoate (VI) (Gowda, Tokarčík *et al.*, 2008). The central –O—C=O group makes a dihedral angle of 6.1 (1)° with the benzoyl ring and 54.9 (1)° with the disubstituted phenyl ring. The two benzene rings make the dihedral angle of 49.1 (1)°, compared to the values of 55.7° for (II), 76.0 (1)° (III), 73.04 (8)° (IV), 63.57 (5)° (V) and 80.25 (5)° (VI). There are no classical hydrogen bonds in the crystal structure. The packing of molecules as viewed along the *b* axis is shown in Fig.2. The long axes of the molecules are directed along the *c* axis.

### Experimental

The title compound was prepared according to the literature method (Nayak & Gowda, 2009). The purity of the compound was checked by determining its melting point. It was characterized by recording its infrared and NMR spectra (Nayak & Gowda, 2009). Colorless Single crystals of the title compound were obtained by slow evaporation of its ethanol solution. The X-ray diffraction studies were made at room temperature.

### Refinement

H atoms were placed in calculated positions and subsequently constrained to ride on their parent atoms, with C–H distances of 0.93 Å (C-aromatic) and 0.96 Å (*C*-methyl). The *U*_iso_(H) values were set at 1.2 *U*_eq_(C aromatic) and 1.5 *U*_eq_(C methyl). The C15 methyl group exhibits orientational disorder of the   H atoms, which were treated using the *SHELX* instruction AFIX 127.

### Crystal data

**Table:**

C_16_H_16_O_2_	*F*(000) = 512
*M**_r_* = 240.29	*D*_x_ = 1.212 Mg m^−^^3^
Monoclinic, *P*2_1_/*n*	Mo *K*α radiation, λ = 0.71073 Å
Hall symbol: -P 2yn	Cell parameters from 8238 reflections
*a* = 11.8022 (3) Å	θ = 2.6–29.1°
*b* = 7.4959 (2) Å	µ = 0.08 mm^−^^1^
*c* = 15.6288 (4) Å	*T* = 295 K
β = 107.760 (3)°	Block, colourless
*V* = 1316.75 (6) Å^3^	0.52 × 0.38 × 0.12 mm
*Z* = 4	

### Data collection

**Table:**

Oxford Diffraction Xcalibur2 diffractometer with a Sapphire CCD detector	2497 independent reflections
graphite	1917 reflections with *I* > 2σ(*I*)
Detector resolution: 10.434 pixels mm^-1^	*R*_int_ = 0.018
ω scans	θ_max_ = 25.7°, θ_min_ = 2.6°
Absorption correction: multi-scan (*CrysAlis RED*; Oxford Diffraction, 2009)	*h* = −14→14
*T*_min_ = 0.96, *T*_max_ = 0.991	*k* = −9→9
15897 measured reflections	*l* = −19→19

### Refinement

**Table:**

Refinement on *F*^2^	Secondary atom site location: difference Fourier map
Least-squares matrix: full	Hydrogen site location: inferred from neighbouring sites
*R*[*F*^2^ > 2σ(*F*^2^)] = 0.042	H-atom parameters constrained
*wR*(*F*^2^) = 0.126	*w* = 1/[σ^2^(*F*_o_^2^) + (0.0673*P*)^2^ + 0.1263*P*] where *P* = (*F*_o_^2^ + 2*F*_c_^2^)/3
*S* = 1.09	(Δ/σ)_max_ < 0.001
2497 reflections	Δρ_max_ = 0.15 e Å^−^^3^
167 parameters	Δρ_min_ = −0.14 e Å^−^^3^
0 restraints	Extinction correction: *SHELXL97* (Sheldrick, 2008), Fc^*^=kFc[1+0.001xFc^2^λ^3^/sin(2θ)]^-1/4^
Primary atom site location: structure-invariant direct methods	Extinction coefficient: 0.014 (2)

### Special details

**Table:**

Geometry. All e.s.d.'s (except the e.s.d. in the dihedral angle between two l.s. planes) are estimated using the full covariance matrix. The cell e.s.d.'s are taken into account individually in the estimation of e.s.d.'s in distances, angles and torsion angles; correlations between e.s.d.'s in cell parameters are only used when they are defined by crystal symmetry. An approximate (isotropic) treatment of cell e.s.d.'s is used for estimating e.s.d.'s involving l.s. planes.
Refinement. Refinement of *F*^2^ against ALL reflections. The weighted *R*-factor *wR* and goodness of fit *S* are based on *F*^2^, conventional *R*-factors *R* are based on *F*, with *F* set to zero for negative *F*^2^. The threshold expression of *F*^2^ > σ(*F*^2^) is used only for calculating *R*-factors(gt) *etc*. and is not relevant to the choice of reflections for refinement. *R*-factors based on *F*^2^ are statistically about twice as large as those based on *F*, and *R*- factors based on ALL data will be even larger.

### Fractional atomic coordinates and isotropic or equivalent isotropic displacement parameters (Å^2^)

**Table:**

	*x*	*y*	*z*	*U*_iso_*/*U*_eq_	Occ. (<1)
C1	0.40414 (14)	0.71817 (19)	−0.01638 (9)	0.0553 (4)	
C2	0.51327 (13)	0.76241 (18)	−0.02574 (9)	0.0527 (4)	
C3	0.51869 (13)	0.7825 (2)	−0.11255 (9)	0.0579 (4)	
H3	0.5913	0.8118	−0.1207	0.069*	
C4	0.42059 (14)	0.7608 (2)	−0.18776 (9)	0.0604 (4)	
C5	0.31413 (15)	0.7133 (2)	−0.17447 (10)	0.0701 (5)	
H5	0.2473	0.6958	−0.224	0.084*	
C6	0.30490 (14)	0.6913 (2)	−0.08916 (10)	0.0670 (4)	
H6	0.2328	0.6589	−0.081	0.08*	
C7	0.33024 (12)	0.79735 (19)	0.10460 (10)	0.0550 (4)	
C8	0.36066 (12)	0.78320 (18)	0.20315 (9)	0.0515 (4)	
C9	0.45901 (12)	0.6895 (2)	0.25536 (9)	0.0563 (4)	
H9	0.5079	0.6299	0.2281	0.068*	
C10	0.48429 (13)	0.6843 (2)	0.34706 (9)	0.0602 (4)	
H10	0.5504	0.6208	0.381	0.072*	
C11	0.41410 (14)	0.77096 (19)	0.39020 (10)	0.0593 (4)	
C12	0.31643 (14)	0.8645 (2)	0.33777 (11)	0.0669 (4)	
H12	0.2677	0.9238	0.3653	0.08*	
C13	0.28975 (13)	0.8717 (2)	0.24585 (10)	0.0632 (4)	
H13	0.2239	0.9361	0.2121	0.076*	
C14	0.62123 (14)	0.7885 (2)	0.05441 (10)	0.0680 (4)	
H14A	0.6063	0.8821	0.0915	0.102*	
H14B	0.6879	0.8203	0.0346	0.102*	
H14C	0.6384	0.6798	0.0884	0.102*	
C15	0.43107 (18)	0.7894 (3)	−0.28053 (10)	0.0820 (5)	
H15A	0.5134	0.8025	−0.2767	0.123*	0.5
H15B	0.3884	0.8953	−0.3062	0.123*	0.5
H15C	0.3981	0.6886	−0.3177	0.123*	0.5
H15D	0.3532	0.7884	−0.3237	0.123*	0.5
H15E	0.4782	0.6957	−0.2942	0.123*	0.5
H15F	0.4685	0.9023	−0.2827	0.123*	0.5
C16	0.44110 (17)	0.7610 (2)	0.49069 (11)	0.0781 (5)	
H16A	0.4014	0.6596	0.5059	0.117*	
H16B	0.4138	0.8679	0.512	0.117*	
H16C	0.5254	0.7492	0.5183	0.117*	
O1	0.40148 (9)	0.69351 (15)	0.07226 (6)	0.0647 (3)	
O2	0.25305 (10)	0.88935 (17)	0.05767 (7)	0.0784 (4)	

### Atomic displacement parameters (Å^2^)

**Table:**

	*U*^11^	*U*^22^	*U*^33^	*U*^12^	*U*^13^	*U*^23^
C1	0.0661 (9)	0.0529 (8)	0.0450 (8)	0.0106 (7)	0.0143 (6)	0.0031 (6)
C2	0.0564 (8)	0.0514 (8)	0.0461 (8)	0.0135 (6)	0.0092 (6)	0.0001 (6)
C3	0.0614 (9)	0.0608 (9)	0.0507 (8)	0.0127 (7)	0.0157 (7)	0.0010 (6)
C4	0.0715 (10)	0.0599 (9)	0.0454 (8)	0.0123 (7)	0.0111 (7)	−0.0027 (6)
C5	0.0696 (10)	0.0784 (11)	0.0503 (9)	0.0004 (8)	0.0004 (7)	−0.0058 (7)
C6	0.0607 (9)	0.0752 (11)	0.0612 (10)	−0.0030 (7)	0.0125 (7)	−0.0009 (8)
C7	0.0523 (8)	0.0551 (9)	0.0580 (8)	0.0018 (6)	0.0176 (6)	0.0054 (6)
C8	0.0521 (7)	0.0507 (8)	0.0537 (8)	−0.0020 (6)	0.0191 (6)	0.0046 (6)
C9	0.0560 (8)	0.0596 (9)	0.0561 (8)	0.0043 (6)	0.0215 (6)	0.0064 (7)
C10	0.0592 (8)	0.0648 (9)	0.0568 (9)	0.0011 (7)	0.0179 (7)	0.0098 (7)
C11	0.0710 (9)	0.0559 (9)	0.0548 (8)	−0.0110 (7)	0.0247 (7)	0.0020 (7)
C12	0.0757 (10)	0.0668 (10)	0.0682 (10)	0.0058 (8)	0.0367 (8)	0.0008 (8)
C13	0.0616 (8)	0.0649 (10)	0.0663 (9)	0.0112 (7)	0.0245 (7)	0.0082 (7)
C14	0.0628 (9)	0.0794 (11)	0.0526 (9)	0.0122 (8)	0.0041 (7)	0.0054 (7)
C15	0.0993 (13)	0.0965 (14)	0.0477 (9)	0.0158 (10)	0.0185 (9)	0.0004 (8)
C16	0.1006 (13)	0.0819 (12)	0.0553 (9)	−0.0093 (10)	0.0287 (9)	0.0016 (8)
O1	0.0757 (7)	0.0698 (7)	0.0500 (6)	0.0191 (5)	0.0213 (5)	0.0094 (5)
O2	0.0729 (7)	0.0965 (9)	0.0624 (7)	0.0288 (6)	0.0154 (5)	0.0131 (6)

### Geometric parameters (Å, °)

**Table:**

C1—C6	1.376 (2)	C10—C11	1.380 (2)
C1—C2	1.380 (2)	C10—H10	0.93
C1—O1	1.4075 (16)	C11—C12	1.384 (2)
C2—C3	1.3858 (19)	C11—C16	1.506 (2)
C2—C14	1.5023 (19)	C12—C13	1.375 (2)
C3—C4	1.385 (2)	C12—H12	0.93
C3—H3	0.93	C13—H13	0.93
C4—C5	1.381 (2)	C14—H14A	0.96
C4—C15	1.508 (2)	C14—H14B	0.96
C5—C6	1.380 (2)	C14—H14C	0.96
C5—H5	0.93	C15—H15A	0.96
C6—H6	0.93	C15—H15B	0.96
C7—O2	1.1982 (16)	C15—H15C	0.96
C7—O1	1.3519 (17)	C15—H15D	0.96
C7—C8	1.475 (2)	C15—H15E	0.96
C8—C13	1.388 (2)	C15—H15F	0.96
C8—C9	1.3889 (19)	C16—H16A	0.96
C9—C10	1.373 (2)	C16—H16B	0.96
C9—H9	0.93	C16—H16C	0.96
			
C6—C1—C2	122.28 (14)	C12—C13—H13	119.9
C6—C1—O1	121.70 (14)	C8—C13—H13	119.9
C2—C1—O1	115.94 (13)	C2—C14—H14A	109.5
C1—C2—C3	116.94 (13)	C2—C14—H14B	109.5
C1—C2—C14	121.63 (13)	H14A—C14—H14B	109.5
C3—C2—C14	121.43 (14)	C2—C14—H14C	109.5
C4—C3—C2	122.82 (15)	H14A—C14—H14C	109.5
C4—C3—H3	118.6	H14B—C14—H14C	109.5
C2—C3—H3	118.6	C4—C15—H15A	109.5
C5—C4—C3	117.76 (14)	C4—C15—H15B	109.5
C5—C4—C15	121.77 (14)	H15A—C15—H15B	109.5
C3—C4—C15	120.46 (15)	C4—C15—H15C	109.5
C6—C5—C4	121.29 (14)	H15A—C15—H15C	109.5
C6—C5—H5	119.4	H15B—C15—H15C	109.5
C4—C5—H5	119.4	C4—C15—H15D	109.5
C1—C6—C5	118.88 (15)	H15A—C15—H15D	141.1
C1—C6—H6	120.6	H15B—C15—H15D	56.3
C5—C6—H6	120.6	H15C—C15—H15D	56.3
O2—C7—O1	123.10 (13)	C4—C15—H15E	109.5
O2—C7—C8	125.31 (13)	H15A—C15—H15E	56.3
O1—C7—C8	111.59 (12)	H15B—C15—H15E	141.1
C13—C8—C9	118.54 (13)	H15C—C15—H15E	56.3
C13—C8—C7	118.51 (13)	H15D—C15—H15E	109.5
C9—C8—C7	122.93 (13)	C4—C15—H15F	109.5
C10—C9—C8	120.28 (14)	H15A—C15—H15F	56.3
C10—C9—H9	119.9	H15B—C15—H15F	56.3
C8—C9—H9	119.9	H15C—C15—H15F	141.1
C9—C10—C11	121.70 (14)	H15D—C15—H15F	109.5
C9—C10—H10	119.1	H15E—C15—H15F	109.5
C11—C10—H10	119.1	C11—C16—H16A	109.5
C10—C11—C12	117.70 (14)	C11—C16—H16B	109.5
C10—C11—C16	121.25 (15)	H16A—C16—H16B	109.5
C12—C11—C16	121.04 (14)	C11—C16—H16C	109.5
C13—C12—C11	121.51 (14)	H16A—C16—H16C	109.5
C13—C12—H12	119.2	H16B—C16—H16C	109.5
C11—C12—H12	119.2	C7—O1—C1	119.70 (11)
C12—C13—C8	120.27 (14)		
			
C6—C1—C2—C3	1.2 (2)	O1—C7—C8—C9	6.60 (19)
O1—C1—C2—C3	178.26 (12)	C13—C8—C9—C10	0.4 (2)
C6—C1—C2—C14	−179.04 (14)	C7—C8—C9—C10	178.54 (13)
O1—C1—C2—C14	−2.0 (2)	C8—C9—C10—C11	−0.1 (2)
C1—C2—C3—C4	0.3 (2)	C9—C10—C11—C12	−0.1 (2)
C14—C2—C3—C4	−179.44 (14)	C9—C10—C11—C16	178.61 (14)
C2—C3—C4—C5	−1.5 (2)	C10—C11—C12—C13	−0.1 (2)
C2—C3—C4—C15	178.28 (14)	C16—C11—C12—C13	−178.80 (15)
C3—C4—C5—C6	1.2 (2)	C11—C12—C13—C8	0.4 (2)
C15—C4—C5—C6	−178.55 (15)	C9—C8—C13—C12	−0.6 (2)
C2—C1—C6—C5	−1.5 (2)	C7—C8—C13—C12	−178.81 (14)
O1—C1—C6—C5	−178.35 (14)	O2—C7—O1—C1	13.5 (2)
C4—C5—C6—C1	0.2 (3)	C8—C7—O1—C1	−166.00 (12)
O2—C7—C8—C13	5.2 (2)	C6—C1—O1—C7	−62.88 (19)
O1—C7—C8—C13	−175.27 (13)	C2—C1—O1—C7	120.07 (15)
O2—C7—C8—C9	−172.89 (15)		
